# Involvement of metabotropic glutamate receptor 5, AKT/PI3K Signaling and NF-κB pathway in methamphetamine-mediated increase in IL-6 and IL-8 expression in astrocytes

**DOI:** 10.1186/1742-2094-9-52

**Published:** 2012-03-15

**Authors:** Ankit Shah, Peter S Silverstein, Dhirendra P Singh, Anil Kumar

**Affiliations:** 1 Division of Pharmacology, UMKC-School of Pharmacy, Kansas City MO 64108, USA; 2 Department of Ophthalmology and Visual Sciences, Univ. of Nebraska Medical Center, Omaha 68198, USA; 3 Division of Pharmacology, UMKC-School of Pharmacy, 2464 Charlotte Street, Kansas City MO 64108, USA

**Keywords:** gp120, IL-8, Astrocytes, NF-kB, siRNA

## Abstract

Methamphetamine (MA) is one of the commonly used illicit drugs and the central nervous system toxicity of MA is well documented. The mechanisms contributing to this toxicity have not been fully elucidated. In this study, we investigated the effect of MA on the expression levels of the proinflammatory cytokines/chemokines, IL-6 and IL-8 in an astrocytic cell line. The IL-6 and IL-8 RNA levels were found to increase by 4.6 ± 0.2 fold and 3.5 ± 0.2 fold, respectively, after exposure to MA for three days. Exposure of astrocytes to MA for 24 hours also caused increased expression of IL-6 and IL-8 at the level of both RNA and protein. The potential involvement of the nuclear factor-Kappa B (NF-κB) pathway was explored as one of the possible mechanism(s) responsible for the increased induction of IL-6 and IL-8 by MA. The MA-mediated increases in IL-6 and IL-8 were significantly abrogated by SC514. We also found that exposure of astrocytes to MA results in activation of NF-κB through the phosphorylation of IκB-α, followed by translocation of active NF-κB from the cytoplasm to the nucleus. In addition, treatment of cells with a specific inhibitor of metabotropic glutamate receptor-5 (mGluR5) revealed that MA-mediated expression levels of IL-6 and IL-8 were abrogated by this treatment by 42.6 ± 5.8% and 65.5 ± 3.5%, respectively. Also, LY294002, an inhibitor of the Akt/PI3K pathway, abrogated the MA-mediated induction of IL-6 and IL-8 by 77.9 ± 6.6% and 81.4 ± 2.6%, respectively. Thus, our study demonstrates the involvement of an NF-κB-mediated signaling mechanism in the induction of IL-6 and IL-8 by MA. Furthermore, we showed that blockade of mGluR5 can protect astrocytes from MA-mediated increases of proinflammatory cytokines/chemokines suggesting mGluR5 as a potential therapeutic target in treating MA-mediated neurotoxicity.

## Introduction

Methamphetamine (MA) is a psychostimulant in the amphetamine class of drugs and is one of the most commonly abused agents by illicit-drug users. The effects of MA are primarily attributed to its action on dopamine (DA) receptors and transporters [1,2]. Furthermore, the interaction of MA with DA receptors and transporters has been shown to be associated with oxidative stress, which is among the several different mechanisms believed to be responsible for the central nervous system (CNS) toxicity associated with MA [3-5]. In addition to oxidative stress, MA has been shown to increase mitochondrial dysfunction, excitotoxicity [6], blood brain barrier (BBB) damage [6-8] and monocyte infiltration into the CNS [9] along with increased levels of inflammatory markers such as IL-6 and TNF-α [10].

The proinflammatory cytokines/chemokines IL-6 and IL-8 are among the inflammatory responses associated with various neurological disorders including Parkinson's disease [11], Alzheimer's disease [12], and amyotrophic lateral sclerosis (ALS) [13]. A single high dose of MA has been shown to induce IL-6 and TNF-α in the striatum and hippocampus of mice [14,15] and IL-1β in the hypothalamus of rats [16]. However, the specific molecular mechanism(s) involved in the increased expression of these proinflammatory cytokines is still unknown. It is generally accepted that MA induces oxidative stress, which can increase proinflammatory cytokines by increasing the activities of transcription factors such as nuclear factor-Kappa B (NF-κB), activator protein-1 (AP-1) and the cAMP-response element-binding protein (CREB) [17,18]. Furthermore, the role of dopamine receptors and transporters in MA-mediated oxidative stress and neuroinflammation has been extensively investigated [19,20]. A more direct cytotoxic role of MA has been demonstrated to be mediated by the c-Jun N-terminal kinases/mitogen-activated protein kinase (JNK-MAPK) pathway followed by the activation of caspases and the induction of apoptosis [21]. However, the role of astrocytes has been relatively unexplored in terms of the regulation of inflammatory cytokines and the mechanisms underlying MA-mediated expression of proinflammatory cytokines.

Low levels of cytokines and chemokines are constitutively expressed by microglia in the CNS and these can be induced to higher levels by inflammatory mediators [22,23]. However, astrocytes constitute the major cell type present in the brain. Astrocytes are also involved in regulation of numerous pro- and anti-inflammatory cytokines [24]. Oxidative stress in astrocytes is found to be mediated via Akt/PI3K, Nrf2 and NF-κB pathways [25]. Increased inflammatory markers released from astrocytes is associated with a variety of CNS complications such as Alzheimer's disease [26], multiple sclerosis, glaucoma [27] and Parkinson's disease [28]. Furthermore, astrocyte activation has been shown to be critical in the regulation of the rewarding effects induced by drugs of abuse [29]. Thus, it is important to consider the role of astrocytes in neuroinflammatory signaling induced by MA. Our present study was undertaken to address whether MA induced proinflammatory cytokines in astrocytes and to determine the mechanisms responsible for MA-mediated expression of these cytokines.

## Materials and methods

### Cell culture and reagents

All the experiments were performed using SVGA, a clone of SVG astrocytes [30]. The cells were cultured at 37°C in a humidified chamber with 5% CO_2 _in Dulbecco's Modified Eagle's Medium supplemented with 10% FBS, 1% L-glutamine, 1% sodium bicarbonate, 1% non-essential amino acids and 0.1% gentamicin. The cells were allowed to adhere overnight before any treatment. All the experiments lasted for three days and were performed in T-75 flasks. For MA treatments the cells were treated once a day with the drug.

MA and MPEP (an mGluR5 antagonist) were obtained from Sigma (Sigma-Aldrich, St. Louis, MO, US). SC514 and LY294002 were obtained from Cayman chemicals (Cayman Chemicals, Ann Arbor, MI, US). The antagonist treatment was given 1 hour prior to the treatments with MA every day. Specific antibodies against Phospho-IκB-α (Ser32) (14D4), β Tubulin (D-10), Actin (C-2), IκB-α, Lamin B (C-20) and NF-κB p50 (H-119) were obtained from Santa Cruz Biotechnology (Santa Cruz, CA, US).

### Real time RT PCR

**Figure 1:**
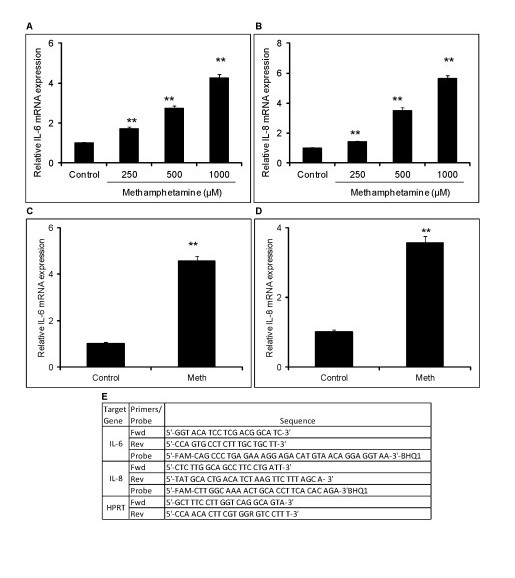
**Methamphetamine increases the expression of IL-6 and IL-8 in astrocytes**. 0.8 × 10^6 ^SVGA astrocytes were treated with different doses of MA for 24 hours and total mRNA was isolated. The relative expression levels of IL-6 (**A**) and IL-8 (**B**) were measured using real time RT-PCR as compared to untreated control. 0.8 × 10^6 ^SVGA were treated with 500 μM of MA for three days in flasks and the cells were harvested 24 hours after the last dose. Total mRNA was isolated and the relative levels of IL-6 (**C**) and IL-8 (**D**) were determined using real time RT-PCR as compared with untreated control. (**E**) Primer sequences used in order to measure the mRNA expression of IL-6 and IL-8 using real time RT-PCR. Each bar represents the mean ± SE of three experiments with each experiment performed in triplicate. The statistical significance was calculated using student's *t*-test and ** denotes *P *≤0.01. MA, methamphetamine.

The mRNA expression levels of IL-6 and IL-8 were quantified by amplifying the total RNA obtained after the treatments. The cells were harvested and total RNA was isolated using an RNeasy mini kit (QIAGEN, Valencia, CA, US) according to the manufacturer's protocol. RNA was then reverse transcribed using RT enzyme for 30 minutes at 50°C. The reverse transcribed product was then amplified for IL-6 and IL-8 and Hypoxanthine-guanine phosphoribosyl transferase (HPRT) with the forward primers, reverse primers and probes as shown in Figure 1E. Briefly, the reverse transcribed cDNA were denatured at 95°C for 15 minutes and amplified for 45 cycles (95°C for 15 seconds, 54°C for 30 seconds and 76°C for 30 seconds). The Ct values for IL-6 and IL-8 were normalized using HPRT expression levels and the fold change in expression levels were calculated using the 2^-ΔΔCt ^method as discussed in [31].

### Immunoblot

SVGA astrocytes were harvested and lysed using RIPA buffer (Boston BioProducts, Ashland, MA, US) upon termination of the treatments to obtain whole cell lysates. Nuclear and cytoplasmic extracts of the cells were prepared using a NE-PER Nuclear Extraction kit (Thermo Fisher, Rockford, IL, US) according to the manufacturer's protocol. The proteins were separated and visualized using western blot analysis as mentioned previously [32].

### Cell viability assay

SVGA astrocytes were enzymatically isolated in the culture medium and stained with 0.4% W/V trypan blue. The total cell count was obtained using a hemocytometer chamber by excluding the dead cells which were stained with the dye.

### Immunocytochemistry

SVGA cells (4 × 10^5^) were cultured on glass cover slips in a 12-well plate and treated with 500 μM of MA. The culture media was supplemented with 1 mg/ml GolgiStop™ (BD Biosciences, San Jose, CA, US) 6 hours prior to termination of the incubation in order to prevent the release of the cytokines. After 24 hours of incubation with MA the cells were fixed with 1:1 ice-cold methanol for 20 minutes at -20°C. The cells were rinsed three times with cold PBS and permeabilized with PBS containing 0.1% Triton X-100 (PBST). The cells were washed three times with PBS followed by blocking with 1% BSA in 0.1% PBST for 30 minutes at room temperature. All the antibodies were diluted in blocking buffer. After blocking, the cells were incubated with either rabbit anti-IL-6 (1:500) or rabbit anti-IL-8 (1:500) and mouse MAb anti-GFAP (GH5) (1:1500) at 4°C overnight in a humidified chamber. After three washes for 5 minutes each with PBS, the cells were incubated in the dark for 1 hour at room temperature with blocking buffer containing either anti-mouse antibody conjugated with Alexa Fluor 555 (1:1000), or anti-rabbit antibody conjugated with Alexa Fluor 488. Finally, the cells were washed three times with PBS for 5 minutes each and mounted on a slide with 10 μl of Vectashield mounting medium with DAPI (Vector Laboratories, Burlingame, CA, US). The fluorescence was observed using a fluorescent Nikon Eclipse E800 confocal microscope (Nikon Instruments, Melville, NY, US). The images were captured using a 60× zoom lens.

### Statistical analysis

All the data are expressed as mean ± SE of three independent experiments with each experiment done in triplicate. The statistical significance was calculated using Student's *t*-test and a significant value of *P *< 0.05 was considered to be statistically significant.

## Results

### Methamphetamine increases the expression of proinflammatory cytokines/chemokines in astrocytes

The neurotoxic effects of MA have been attributed to its potential for inducing oxidative stress through dopamine (DA) receptor and dopamine transporter (DT) dependent mechanisms reviewed in [33-35]. We wished to determine whether MA could increase the expression of proinflammatory cytokines/chemokines such as IL-6 and IL-8 in astrocytes. SVGA astrocytes were treated with 250 μM, 500 μM and 1000 μM of MA for 24 hours. The doses of MA were physiologically relevant based on the levels of MA found in post-mortem brain samples from MA abusers (0.8 to 1 mM) [36]. The mRNA expression levels of IL-6 and IL-8 showed a dose-dependent increase. The MA-induced expression of IL-6 and IL-8 was found to be 1.7 ± 0.1, 2.7 ± 0.1, and 4.2 ± 0.2 fold and 1.4 ± 0.1, 3.5 ± 0.2 and 5.6 ± 0.2 fold, respectively for 250 μM, 500 μM and 1000 μM of MA, respectively (Figure 1A, B). Furthermore, we also wanted to determine the effect of chronic exposure of MA on astrocytes. We treated the astrocytes with 500 μM MA for three days, once a day, as the dose is relevant to the levels found physiologically in MA abusers [36]. The chronic treatment with MA resulted in increased expression of IL-6 and IL-8 by 4.6 ± 0.2 fold and 3.5 ± 0.2 fold, respectively (Figure 1C, D). These results clearly indicate that MA can increase the expression of proinflammatory cytokines/chemokines such as IL-6 and IL-8.

### Methamphetamine increases intracellular levels of IL-6 and IL-8

**Figure 2:**
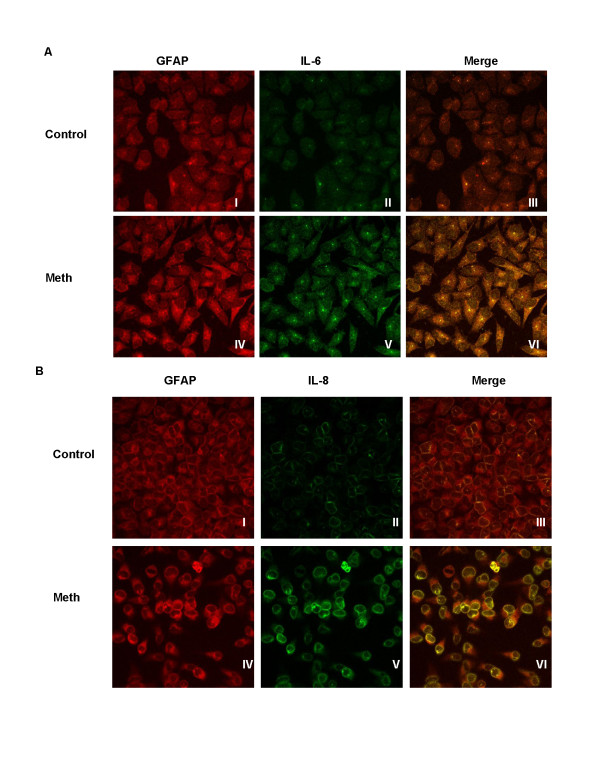
**Methamphetamine treatment increased the expression of IL-6 and IL-8 in the astrocytes**. SVGA astrocytes were grown on coverslips and were treated with 500 μM of methamphetamine (MA) for 24 hours followed by methanol-fixation. Golgi-Stop™ was added 6 hours prior to fixing in order to block the release of the cytokines from the cells into the supernatants. The coverslips were blocked, and then incubated with primary antibody for IL-6/IL-8 and **Glial Fibrillary Acidic Protein **(**GFAP) **and secondary antibodies conjugated with Alexafluor488 to detect IL-6 and IL-8 and an antibody conjugated with Alexafluor555 to detect GFAP. The coverslips were then mounted using medium containing DAPI and were observed under a confocal microscope to determine the localization and expression of IL-6 (A) and IL-8 (B). Untreated cells were stained individually for IL-6, IL-8 and GFAP and did not show any non-specific fluorescence (data not shown).

After determining that MA increased levels of IL-6 and IL-8 mRNA we wanted to confirm that increased levels of RNA were reflected in increased production of IL-6 and IL-8 protein. SVGA astrocytes were treated with 500 μM MA for 24 hours and intracellular levels of IL-6 and IL-8 proteins were observed using immunhistochemistry. Although in the control cells there were only minor amounts of IL-6 or IL-8 proteins, in cells treated with MA for 24 hours increased levels of Il-6 and IL-8 proteins were clearly visible (Figure 2).

### Methamphetamine-mediated induction of proinflammatory cytokines/chemokines is mediated via NF-κB pathway

**Figure 3:**
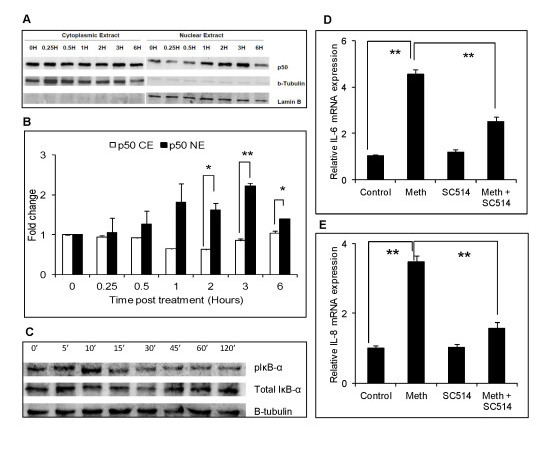
**Methamphetamine induction of IL-6 and IL-8 is mediated via NF-κB pathway**. (**A**) The proteins from the nucleus and cytoplasm of SVGA cells were collected after various time-periods as indicated and the translocation of p50 was measured by comparing the expression in the nucleus and cytoplasm. The expression in the nucleus and cytoplasm were normalized with laminB and β-Tubulin, respectively, as housekeeping genes. (**B**) The bar chart represents the mean ± SE of three independent experiments and the blot is a representation of three experiments. (**C**) SVGA cells were treated with 500 μM of MA for the specified time and the total cell lysates were collected as mentioned in Materials and Methods. Total IκB-α was used as an experimental control and actin was used as a loading control. The blot is representative of three independent experiments. SVGA astrocytes were treated with 10 μM of SC514 for one hour prior to MA treatment every day for three days and the total mRNA was isolated 24 hours after the last dose. The expression levels of IL-6 and IL-8 were measured with real time RT-PCR and the percent mRNA expression of IL-6 (**D**) and IL-8 (**E**) were calculated relative to MA-mediated expression levels. SC514-treated cells did not alter the basal expression levels of IL-6 and IL-8 (data not shown). Each bar represents the mean ± SE of three experiments with each experiment performed in triplicate. The statistical significance was calculated using student's *t*-test and * and ** denotes *P *≤ 0.05 and ≤ 0.01, respectively. MA, methamphetamine.

We then wished to determine the signaling mechanism involved in MA-mediated increased expression of IL-6 and IL-8. The potential involvement of the NF-κB pathway was investigated because the promoters for both IL-6 and IL-8 contain a binding site for NF-κB and this transcription factor is known to be involved in neurological disorders associated with increased inflammation [37-40]. In order to determine whether MA can induce NF-κB activation and translocation, SVGA astrocytes were treated with 500 μM MA over a time period of 0 to 6 hours. The cytoplasmic and nuclear fractions of the astrocytes were collected and the levels of p50 in cytoplasmic and nuclear fractions were measured in MA treated cells and compared to untreated controls (Figure 3A, B). Clearly, MA-treated cells showed a time-dependent increase in p50 translocation from the cytoplasm to the nucleus, with the greatest translocation observed at 3 hours (2.2 ± 0.1 fold).

In order to further confirm these results we measured the phosphorylated form of IκB-α in the whole cell lysates of the astrocytes treated with 500 μM of MA over a period of 0 to 120 minutes. Consistent with the previous results, MA treatment was found to increase the phosphorylation of IκB-α, as evidenced by the increased levels of p-IκB-α in MA-treated astrocytes at 10 minutes (Figure 3C).

We also evaluated the effect of SC514, a specific inhibitor of inhibitory Kappa B kinase (IKK) on the expression levels of IL-6 and IL-8 in MA-treated astrocytes. The cells were treated with 10 μM SC514 (IC50 = 14.5 μM) one hour prior to each MA treatment over the three-day course of the experiment. The concentration of the antagonist was determined based on the IC50 value of the SC-514 and the viability of the cells, which was found to be approximately 90% at the concentration that was used (data not shown). The mRNA expression levels of IL-6 and IL-8 were measured using real time RT-PCR and the percent inhibition of MA-mediated expression levels of IL-6 and IL-8 were found to be 56.7 ± 5.1% and 78.4 ± 7.8%, respectively (Figure 3D, E). Together, all these results strongly suggest that induction of IL-6 and IL-8 by MA involves the activation and translocation of NF-κB.

### Role of mGluR5 and Akt/PI3K in MA-mediated expression of proinflammatory cytokines/chemokines

**Figure 4:**
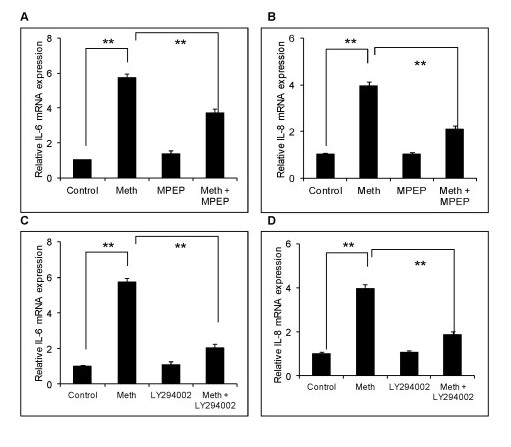
**Role of mGluR5 and Akt/PI3K in MA-mediated expression of IL-6 and IL-8**. SVGA astrocytes were treated with 25 μM of MPEP or 10 μM of LY294002 for 1 hour prior to the MA treatment every day for three days and the total mRNA was isolated 24 hours after the last treatment of MA. The relative mRNA expression levels were measured for IL-6 and IL-8 using real time RT-PCR and the % mRNA expression levels of IL-6 and IL-8 due to MPEP (**A**, **B**, respectively) and LY294002 (**C**, **D**, respectively) were determined relative to cytokine levels measured from SVGA cells treated with MA in the absence of the inhibitors. Cells treated with inhibitors alone did not alter the basal expression levels of IL-6 and IL-8 (data not shown). Cytokine expression levels of MA-treated cells relative to untreated controls were similar to those levels shown in Figure 1C and D. Each bar represents the mean ± SE of three experiments with each experiment performed in triplicate. The statistical significance was calculated using student's *t*-test and ** denotes *P *≤ 0.01. mGluR5, metabotropic glutamate receptor-5; MA, methamphetamine.

Having established the role of the NF-κB pathway in the induction of IL-6 and IL-8 by MA, we were interested in the receptor involved in mediating this response. There is an extensive body of literature demonstrating that many of the effects of MA are mediated through the DA receptor/DA transporter (DT). However, recent reports also suggest the possible involvement of metabotropic glutamate receptors in MA-mediated neurotoxicity and/or cognitive impairment [41-43]. In order to determine whether the metabotropic glutamate receptor-5 (mGluR5) plays a role in the MA-mediated increase in IL-6 and IL-8 levels in astrocytes, we treated the astrocytes with 25 μM of MPEP, a specific inhibitor of mGluR5, one hour prior to MA treatment for three days once in a day (o.i.d.). The dose of MPEP was determined based on the cell viability as observed by trypan blue staining (data not shown). We observed that MPEP abrogated the MA-induced expression levels of IL-6 and IL-8 by 42.6 ± 5.8% and 58.1 ± 2.9%, respectively (Figure 4A, B). Thus, these results clearly indicate an interaction of the mGluR5 receptor with MA, which leads to increased expressions of proinflammatory cytokines/chemokines.

In order to determine the downstream signaling cascade leading to the NF-κB pathway, we investigated the potential role of the Akt/PI3K pathway. The metabotropic glutamate receptor pathway requires the activation of the Akt/PI3K signaling cascade for its activity [44-46]. Therefore, we treated the astrocytes with 10 μM of LY294002, a specific inhibitor of Akt/PI3K, for 1 hour prior to MA treatment for each of three days. The dose of LY294002 was determined based on the cell viability as observed by trypan blue staining (data not shown). As per our hypothesis, LY294002 did abrogate the MA-mediated expression levels of IL-6 and IL-8 by 77.9 ± 6.6% and 81.4 ± 2.6%, respectively (Figure 4C, D). Thus, these results provide strong evidence for the involvement of the Akt/PI3K signaling mechanism in the induction of IL-6 and IL-8 by MA.

## Discussion

In the CNS, astrocytes are the most abundant cells and they are associated with several functions including metabolic support of neurons and modulation of neurotransmission. In the present study, we investigated the role of astrocytes in neuroinflammation produced by MA. We found that MA treatment of astrocytes can result in increased levels of IL-6 and IL-8 in a dose dependent manner. Furthermore, we demonstrated that the increases in IL-6 and IL-8 expression observed at the level of RNA were consistent with our observation of increased expression of IL-6 and IL-8 proteins. A single dose of MA in mice has been found to be associated with neurotoxicity mediated by increased expression of proinflammatory cytokines such as TNF-α, IL-1β and IL-6 [10,15]. In this study, we investigated the effect of multiple doses of MA on the expression of proinflammatory cytokines in astrocytes. Our results demonstrate that MA can produce neuroinflammation in prolonged treatments. Furthermore, the expression of these cytokines is more pronounced with increasing doses of MA, indicative of increased neurotoxicity at higher doses of MA.

We next investigated the signaling mechanisms responsible for the MA-mediated increases in expression of IL-6 and IL-8. MA-mediated oxidative stress and mitochondrial dysfunction are found to play a major role in its neuroinflammatory effects [6,47,48]. The induction of a neuroinflammatory response due to MA has been shown to be associated with increased activities of certain transcription factors, including STAT1, STAT3, AP1, CREB and NF-κB [17,49]. Since NF-κB has also been found to be associated with multiple neurological disorders and neuroinflammatory complications, we investigated whether NF-κB plays any role in the increased cytokine expression induced by MA. Our studies clearly showed multiple lines of evidence that support our hypothesis that the MA-mediated increase of proinflammatory cytokines/chemokines is dependent on the NF-κB pathway. To our knowledge, this is the first demonstration that MA-induced increases in proinflammatory cytokines/chemokines are mediated through the NF-κB pathway. Thus, the NF-κB pathway appears to be involved in mediating the induction of proinflammatory cytokines/chemokines by MA, as well as in mediating the response to oxidative stress induced by MA. The role of the dopamine receptor and transporters has been extensively studied to address the neuroinflammatory roles of MA in the CNS [33-35]. Recently, however, the effect on the excitatory neurotransmitter via glutamate receptors has also been shown to be responsible for MA-mediated cognitive impairments [43]. Furthermore, inhibition of the metabotropic glutamate receptors, mGluR5 in particular, has been shown to reduce the self-administration of MA in rats [50]. MA-mediated extracellular glutamate is found to augment the excitotoxicity in the striatums of rats [51]. Furthermore, our results in the present study clearly demonstrate that mGluR5 plays a role in the MA-mediated induction of IL-6 and IL-8. Thus, our study suggests a possible link between the oxidative stress and mGluR5 activity since both phenomena are due to MA treatment. Furthermore, recent studies in spinal cord injury and neonatal excitotoxic lesions have suggested the potential utilization of mGluR5 as a therapeutic target [52,53]. Our findings also suggest mGluR5 as a therapeutic target for abrogation of MA-mediated expression of neuroinflammatory cytokines/chemokines. Furthermore, we sought to investigate a link between the effects of MA on mGluR5 and NF-κB activation. Since the classical Huntington's disease pathway involves activation of the Akt/PI3K pathway, which is mediated via the mGluR5 receptor [54], we hypothesized a role for Akt/PI3K in the signaling cascade induced by MA. Our results support this hypothesis, because the antagonist for Akt/PI3K abrogated MA-mediated expression of IL-6 and IL-8.

**Figure 5:**
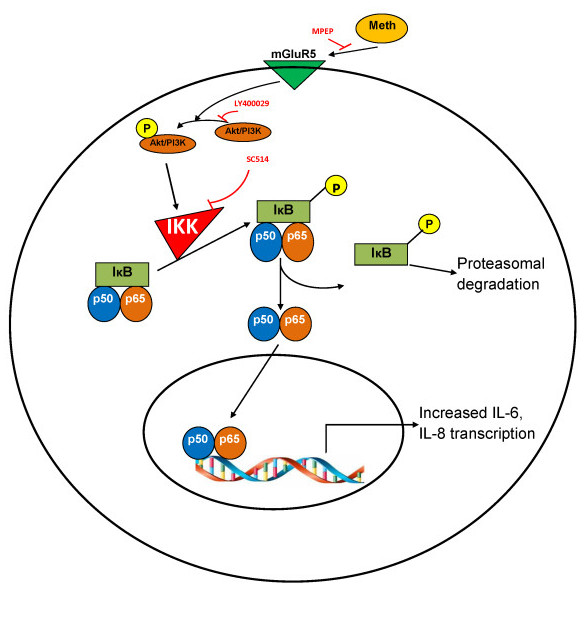
**Schematic of the signaling pathways involved in MA-mediated expression levels of IL-6 and IL-8**. Exposure to MA leads to the activation of excitatory neurotransmitters and leads to mGluR5-mediated activation of Akt/PI3K signaling pathways. Activated Akt/PI3K phosphorylates IKK which results in the phosporylation of p-IκB and releases the heterodimeric NF-κB (p50/p65) in the cytoplasm. NF-κB then translocates from the cytoplasm to the nucleus and leads to enhanced IL-6 and IL-8 expression in astrocytes. IKK, inhibitory Kappa B kinase; MA, methamphetamine; mGluR5, metabotropic glutamate receptor-5; NF-κB, nuclear factor-Kappa B.

In conclusion, we demonstrated that MA could increase the expression of proinflammatory cytokines/chemokines in astrocytes in a dose-dependent manner. MA alters the mGluR5 receptor activity, which activates the Akt/PI3K cascade. This may further activate the phosphorylation of IκB-α via the kinase activity of IKK releasing the free form of the heterodimeric NF-κB (p50-p65). Finally the active NF-κB then translocates from cytoplasm to the nucleus and promotes the transcription of IL-6 and IL-8 (Figure 5). The temporal relationship between the pathways that is presented in Figure 5 is based upon the work presented here and also includes findings from other laboratories. The activation of PI3/Akt has been shown to be mediated through mGluR5 [44,55]. Furthermore, involvement of the NF-κB pathway in the regulation of various pro-inflammatory cytokines/chemokines has been well reported [37-40]. In our study, we demonstrated abrogation of MA-mediated IL-6 expression with the use of both mGluR5 and Akt/PI3K inhibitors. Thus, in addition to the current paradigm presented in the literature, our study provides essential evidence suggesting involvement of these pathways with regard to MA-mediated signaling. Thus, our study provides vital information to understand better the role of MA in the expression of proinflammatory cytokines/chemokines in astrocytes and provides an insight in the development of therapeutic strategies to counteract MA-mediated inflammation.

## Competing interests

The authors declare that they have no competing interests.

## Authors' contributions

AS performed all the experiments and prepared the first draft of the manuscript. PSS analyzed the data and edited the manuscript. DPS helped in the NF-kB experiments. AK designed the project, supervised AS throughout the experimental phase and finalized the manuscript. All authors have read and approved the final manuscript.
